# Barriers to adherence with tuberculosis contact investigation in six provinces of Vietnam: a nested case–control study

**DOI:** 10.1186/s12879-015-0816-0

**Published:** 2015-02-26

**Authors:** Gregory James Fox, Le Phuong Loan, Nguyen Viet Nhung, Nguyen Thi Loi, Dinh Ngoc Sy, Warwick John Britton, Guy Barrington Marks

**Affiliations:** Woolcock Institute of Medical Research, University of Sydney, 431 Glebe Point Road, Glebe, Sydney 2037 Australia; Sydney Medical School, University of Sydney, Sydney, 2006 Australia; National Lung Hospital, 463 Hoang Hoa Tham Street, Ba Dinh, Hanoi, Vietnam; Centenary Institute of Cancer Medicine and Cell Biology, University of Sydney, Missenden Road Camperdown, Sydney, 2050 Australia; South Western Sydney Clinical School, University of New South Wales, Sydney, 2052 Australia

**Keywords:** Tuberculosis, Contact tracing, Screening, Infectious Disease Contact Tracing, Public health, Tuberculosis, Pulmonary, *Mycobacterium tuberculosis*

## Abstract

**Background:**

Close contacts of patients with tuberculosis (TB) have a substantial risk of developing the disease, particularly during the first year after exposure. Household contact investigation has recently been recommended as a strategy to enhance case detection in high-burden countries. However the barriers to its implementation in these settings remain poorly understood.

**Methods:**

A nested case–control study was conducted in Vietnam within the context of a large cluster randomised controlled trial of active screening for TB in household contacts of patients with pulmonary TB. The study population comprised contacts (and their index patients) from 12 Districts in six provinces throughout the country. Cases were contacts (and their index patients) that did not attend the scheduled screening appointment. Controls were those who did attend. We assessed relevant knowledge, attitudes and practices in cases and controls.

**Results:**

The acceptability of contact investigation was high among both cases (n = 109) and controls (n = 194). Both cases (47%) and controls (36%) commonly reported discrimination against people with TB. Cases were less likely than controls to understand that sharing sleeping quarters with a TB patient increased their risk of disease (OR 0.46, 0.27 – 0.78) or recognise TB as an infectious disease (OR 0.65, 0.39 – 1.08). A higher proportion of cases than controls held the mistaken traditional belief that a non-infectious form of TB caused the disease (OR 1.69, 1.02 – 2.78).

**Conclusions:**

The knowledge, attitudes and practices of contacts and TB patients influence their ongoing participation in contact investigation. TB case detection policies in high-prevalence settings can be strengthened by systematically evaluating and addressing locally important barriers to attendance.

**Trial registration:**

Australian New Zealand Clinical Trials Registry, ACTRN12610000600044.

**Electronic supplementary material:**

The online version of this article (doi:10.1186/s12879-015-0816-0) contains supplementary material, which is available to authorized users.

## Background

Tuberculosis (TB) control remains a major global public health challenge, with most of the 9 million people affected each year living in resource-limited countries [1]. Despite the widespread availability of effective therapies for TB, the global average decline in incidence has been only 2% annually. It is estimated that, each year, three million new cases of TB remain undiagnosed [1]. Actively screening high-risk groups for TB could increase the proportion of infectious cases that are diagnosed and treated and, thereby, reduce ongoing transmission of TB in the community [2,3]. Household contacts of patients with TB have a higher risk of developing TB than the general population. A recent meta-analysis of published contact investigation studies showed that, overall, 3.1% (95% CI 2.2-4.4%) of household contacts had TB disease at the time their infectious household member was diagnosed [4]. The study also showed high rates of incident TB over the four years following enrolment, suggesting that serial screening of contacts is a high yield strategy. Evidence from community-based studies also points towards a possible impact upon the burden of TB in high-prevalence settings [5].

Over the past decade, WHO recommended screening for TB in high-risk groups including household contacts [6], child contacts under 5 years [7] and contacts who are HIV infected [8]. National Tuberculosis Programs in high-prevalence settings have sought to apply these policies in their local contexts [9]. However, they have faced substantial challenges in bridging the gap between screening policies and practice [10]. In particular, convincing asymptomatic contacts to attend screening appointments has been difficult. Understanding the barriers to screening from the perspective of patients and their contacts is critical to the development of effective contact investigation strategies [11-13].

This study aimed to characterise knowledge about TB and perceived barriers to participation in contact investigation among household contacts of adult patients with pulmonary TB managed within the Vietnam National Tuberculosis Program.

## Methods

### Setting

Vietnam is a south-east Asian country with a persistently high prevalence tuberculosis (209 prevalent cases of TB per 100 000 population), despite having achieved good treatment outcomes for over a decade [1]. Consequently, enhanced case finding strategies, such as contact investigation, are a priority for Vietnam. The National TB Program has recently adopted a policy of routine symptom screening and preventive therapy for child contacts. However screening of adult contacts is not performed routinely [14].

### Study design

We conducted a nested case–control study between September 2010 and July 2012 at District TB clinics participating in a larger randomised controlled trial (RCT) of contact investigation that is being undertaken in 8 Provinces throughout Vietnam [15]. District TB units are the primary location of TB diagnosis and treatment for the National TB Program, with chest radiography available at most sites. In the main RCT, which is described elsewhere [15], 70 District TB units were randomised to be either the intervention or control group. Over 10,000 contacts of patients with smear positive pulmonary TB were recruited in each group. In the intervention Districts, ‘index’ TB patients were asked to bring all household contacts to their local District TB unit for screening four times in two years: at the time of the patient’s initial diagnosis, then after six, 12 and 24 months.

District TB units that were randomised to the intervention group were eligible for inclusion in the case–control study if they had demonstrated their capacity to recruit contacts during the early phase of the RCT. This was defined as recruiting at least 50% of eligible index patients, based upon case notification data. This approach was chosen to ensure that health system-related factors alone did not drive the non-attendance of contacts. Based on this criterion, 12 of 36 intervention Districts from the six of the eight provinces in the main study were selected. The study population for the present case–control study comprised all contacts within these 12 Districts that had been enrolled in the main study before 30^th^ September 2011. Within this study population, cases were those who did not attend their scheduled six-month follow-up visit (that is, the first scheduled review after enrolment into the main study) and controls were selected at random from among those who did attend this appointment.

### Questionnaire development and data collection

Contacts were interviewed by District TB clinic staff, using a structured questionnaire (Additional file 1), to ask about their knowledge and attitudes relating to contact investigation. The ‘index’ patients of enrolled contacts also completed a questionnaire. All questionnaires were manually checked for completeness and consistency by study staff, and 10% of contacts were randomly re-interviewed by Provincial staff to verify the accuracy of responses.

Knowledge, attitudes and beliefs about TB were measured using a questionnaire that was administered to all selected cases and controls (and their index patients).

The questionnaire was developed based upon a review of the literature, and three focus group discussions including 26 household contacts and their index patients and health care workers involved in a pilot contact investigation study [16]. Discussion themes were identified by a search of relevant literature and consultation with local physicians. Recordings from the discussions were transcribed, coded and analyzed according to the grounded theory method [17]. The final questionnaire was based on themes arising from the discussions, as well as themes identified in published literature relating to knowledge about TB, attitudes towards contact investigation, and compliance with TB screening.

### Data analysis

Qualitative interviews were recorded digitally, transcribed and translated into English before coding and analysis.

Questionnaire responses were independently entered twice using EpiData 3.1 [18]. Groups of contacts were compared using generalized estimating equations (GEE), with clustering at the level of the index patient. Effects were estimated as odds ratios and 95% confidence intervals. Model covariate selection in multivariate analysis was based upon quasi-likelihood under the independence model criteria (QIC) using reverse covariate selection. Data were analyzed using SAS (v9.3, SAS Institute, Cary, NC). Variables with <10% of missing values were imputed using multiple imputation [19], and those with a higher proportion of missing values were excluded.

We estimated that 136 cases and an equal number of controls would yield 80% power to detect a 40% difference in the prevalence of positive responses between cases and controls as significant (at the 5% level).

### Ethics approval and consent

This study was approved by the Human Research Ethics Committee of the University of Sydney, and the Institutional Review Board of the National Lung Hospital, Hanoi, and was conducted in accordance with the principles of the Helsinki Declaration. Study participants provided written informed consent. The main RCT within which this study was nested (the ACT2 study) has been registered in the Australian New Zealand Clinical Trials Registry ACTRN12610000600044.

## Results

### Participant demographics

**Figure 1:**
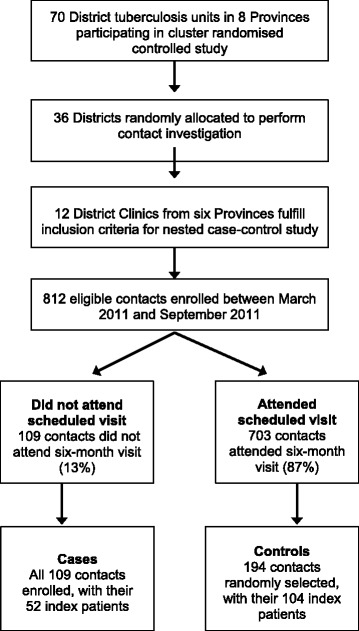
**Consort diagram of study recruitment.**

Among 812 eligible contacts participating in the main RCT in the 12 intervention Districts, all 109 ‘non-attenders’ (cases) and 194 selected ‘attenders’ (controls) agreed to participate in this study (Figure 1). In addition, the 162 index patients of these contacts agreed to complete a questionnaire.

The median age of contact participants was 35 years (interquartile range, IQR, 18 – 50) among cases and 33 years (IQR 20–49) among controls (Table 1) and 60% and 58%, respectively, were female. More cases than controls had previously been treated for TB, although the 95% confidence interval crossed the null (OR 1.36, 0.41 -4.47). Cases were less likely than controls to be from the south of the country than the north (OR 0.43, 0.21 -0.88). The majority of contacts traveled to the clinic by motorbike (84% among cases and 83% among controls). The proportion living more than 10 km from the District TB Unit was similar in cases (28%) and controls (27%).

**Table 1:** **Demographic characteristics of participating household contacts**

**Characteristics**		**Cases** ^**+**^		**Controls** ^*^	
		**N**		**n**	
Total		109		194	
Gender	Female	65	(60%)	113	(58%)
Median age, years (IQR)	35				
		(18–50)	33	(20–49)	
Region of Vietnam	North	19	(17%)	16	(8%)
	Centre	20	(18%)	40	(21%)
	South	70	(64%)	138	(71%)
Setting	Rural	52	(48%)	81	(42%)
	Urban	57	(52%)	113	(58%)
Prior TB history		10	(9%)	10	(5%)
Relationship to TB patient	Parent	17	(16%)	35	(18%)
	Spouse	31	(28%)	59	(30%)
	Child	39	(36%)	64	(33%)
	Sibling	7	(6%)	18	(9%)
	Other relative	15	(14%)	18	(9%)
Highest education level^#^	No schooling	4	(4%)	16	(8%)
	Primary (grades 1–5)	26	(25%)	50	(26%)
	Secondary (grades 6)	29	(28%)	55	(29%)
	High school (grades 7+)	32	(31%)	45	(24%)
	Tertiary	10	(10%)	17	(9%)
	Vocational training	3	(3%)	7	(4%)
Current occupation^¶^	Full time employee	14	(16%)	31	(20%)
	Unemployed	7	(8%)	10	(6%)
	Student	20	(22%)	41	(26%)
	Part time employee	0	(0%)	2	(1%)
	Retired	9	(10%)	9	(6%)
	Self-employed	39	(44%)	63	(40%)
Monthly income^**^	<$25 / month	21	(26%)	32	(23%)
	$25 to $250 / month	52	(64%)	97	(71%)
	> $250 / month	8	(10%)	8	(6%)

*’Cases’ were contacts that did not attend their scheduled appointment. ^+’^Controls’ were contacts that attended their scheduled appointment. ^#^Educational level provided by 104/109 cases and 190/194 controls. ^**¶**^89/94 cases and 156 /194 controls stated their occupation. ^**^81/109 cases and 137/194 controls stated their monthly income.

### Contacts’ attitudes and knowledge about TB and TB screening

There was strong acceptance of TB contact investigation in both groups, with 83% of cases and 88% of controls believing the program to be beneficial (Table 2). Both groups appreciated the increased risk of TB among contacts (70% and 69% respectively), and recognized that TB can severely affect their health (83% and 89% respectively). A perception of discrimination against TB in the community was more common among cases than controls (47% versus 36%, OR 1.55, 0.95 – 2.51, adjusted for region and prior TB). Cases were less likely to understand that TB was caused by an infectious organism (adjusted OR 0.65, 0.39 – 1.08), and were less likely to perceive that sharing a bedroom increased the risk of transmission (adjusted OR 0.46, 0.27 – 0.78). A higher proportion of cases also held a mistaken traditional belief that a non-infectious ‘exhaustible’ form of TB [20] could cause the disease (adjusted OR 1.69, 1.02 – 2.78). Overall, 98% of cases and 97% of controls correctly believed that smoking tobacco was associated with an increased risk of developing TB, however a high proportion incorrectly attributed infection to sharing of utensils (cases 74%, controls 80%) or clothing or towels (61% and 55% respectively). Only 11% and 6% of cases and controls, respectively, believed that Vietnamese traditional medicines alone could cure TB, while most viewed Western medicines as curative (93% in both groups).

**Table 2:** **Perceptions of tuberculosis and tuberculosis screening among contacts**

	**Non-attendee responses (cases)**		**Attendee responses (controls)**		**Odds of non-attendance**	
**Response**	**n**	**(%)**	**n**	**(%)**	**OR**_**adj**_	**95% CI**
Total	109		194			
Contact attitudes towards TB						
Perceive discrimination against TB from outside the family	51	(47%)	70	(36%)	1.55	(0.95 -2.51)
Believe that own risk of TB as being higher than the general population	76	(70%)	134	(69%)	1.2	(0.69 -2.07)
Believe TB screening is beneficial for their family	91	(83%)	171	(88%)	0.55	(0.26 -1.15)
Knowledge and attitudes about TB						
TB can be transmitted by						
Sneezing	41	(38%)	62	(32%)	1.39	(0.84 -2.29)
Talking	79	(72%)	149	(77%)	0.77	(0.44 -1.32)
Sharing utensils	81	(74%)	156	(80%)	0.66	(0.37 -1.16)
Sleeping in the same bedroom	70	(64%)	150	(77%)	0.46	(0.27 -0.78)
Sharing towels, clothes etc.	67	(61%)	107	(55%)	1.25	(0.77 -2.03)
Hugging or kissing	71	(65%)	143	(74%)	0.61	(0.36 -1.03)
Sharing the same toilet	29	(27%)	40	(21%)	1.32	(0.75 -2.31)
Having sexual intercourse	15	(14%)	20	(10%)	1.52	(0.74 -3.13)
TB is caused by*						
An infectious organism	56	(55%)	126	(68%)	0.65	(0.39 -1.08)
Living in an unhygienic environment	85	(83%)	139	(75%)	1.71	(0.91 -3.21)
Inheriting the disease from your parents	33	(32%)	48	(26%)	1.44	(0.84 -2.46)
A form of ‘exhausted TB’ which is not transmissible	64.0	(63%)	93	(50%)	1.69	(1.02 -2.78)
The following people have a higher risk of developing TB*						
Tobacco and bong smokers	100	(98%)	180	(97%)	1.47	(0.29 -7.45)
Children	79	(77%)	141	(76%)	0.99	(0.55 -1.77)
Pregnant women	72	(71%)	138	(74%)	0.8	(0.46 -1.38)
People with weakened immune systems such as diabetes	84	(82%)	154	(83%)	0.9	(0.47 -1.71)
People with poor nutrition	91	(89%)	170	(91%)	0.77	(0.34 -1.74)
Treatment and cure of TB*						
TB can be completely cured if a person takes treatment	102	(100%)	183	(98%)	na	
Traditional medicine can cure TB	12	(12%)	12	(6%)	2.07	(0.89 -4.84)
Western medicine can cure TB	101	(99%)	180	(97%)	4.43	(0.51 -38.82)
TB can severely affect your health	91.0	(89%)	173	(93%)	0.83	(0.34 -2.04)

OR_adj_ – Adjusted odds ratio. CI – Confidence interval. n – number of responses. Adj = adjusted for region and prior TB status. *102 case subjects and 194 control subjects responded.

### Factors affecting attendance at follow-up

When asked about barriers to attending screening 41% of cases identified the distance between the clinic and their house as a barrier to participation (Table 3). 43% of cases found difficulties taking time off work or study to attend the appointment. Significantly more cases than controls believed that once the index patient was screened then there was no need for screening (adjusted OR 2.30, 1.25 -4.24). 70% of cases reported forgetting their scheduled appointment.

**Table 3:** **Explanations by cases for non-attendance at follow-up**

	**n**	**(%)**
**Total**	**105** ^**+**^	
Case responses*		
The distance from my house to the clinic is too far	43	(41%)
I am worried about harmful effects of X-ray	28	(27%)
I prefer to be examined in a private clinic instead	20	(19%)
I am worried about discrimination from other people towards myself and my family	28	(27%)
The patient in my house recovered, so follow-up screening is not necessary	33	(32%)
I initially forgot the scheduled appointment	73	(70%)
The initial screening was negative, so I did not see the need for further screening	25	(24%)
It was time consuming, and difficult to get time off work or study	45	(43%)

^+^The denominator for individual questions differs slightly for each question, based on the number of valid responses. *Indicates an affirmative response.

The index patients of cases were more likely than patients of controls to believe that specific risk groups, namely pregnant women (OR 3.43, 1.53 – 7.70) and people with immune impairment (OR 4.8, 1.37 – 16.83) were more likely to develop TB (Additional file 2: Table S1). The perceptions of stigma, and benefits of screening, were similar in index patients of cases and contacts.

## Discussion

This case–control study, nested within a RCT of serial contact investigation in 70 Districts throughout Vietnam, characterised the knowledge and attitudes of participants towards TB and contact investigation. A substantial proportion of contacts and index patients identified discrimination against people with TB as an important issue, while most believed that screening contacts for TB was beneficial. Cases were less likely to understand TB as an infectious disease, and misunderstandings about the biology of disease and transmission were common.

For the long-term ambition of global TB elimination to be realized [21], new strategies are needed to substantially enhance case-finding and thereby reduce transmission in the community. Consequently, recent WHO policies have provided a framework for the expansion of TB screening in a range of high-risk populations, including household contacts [6,22]. However, if enhanced case-finding strategies are to be successful, international recommendations must be adapted successfully to local clinical contexts [23]. Consequently, it is important that before ‘scaling up’ new screening programs, National TB programs and local health services should consider how local attitudes towards TB screening are likely to influence their success.

This study has identified a number of important misconceptions about the natural history of TB that are likely to affect attendance. One third of cases believed that they were no longer at risk once the TB patient in their household had completed treatment, and one quarter believed an initial negative test was sufficient to exclude subsequent disease. This reflects a lack of understanding about the nature of latent tuberculous infection, which can frequently lead to tuberculosis months or years after the initial exposure [4]. This is likely to reduce motivation to attend serial screening for disease. For contact investigation programs to achieve high rates of re-screening, participants must be educated about their ongoing risk of disease. This finding emphasizes the importance of National TB Programs delivering clear and accurate information to contacts about their ongoing risk of reactivation in the several years after exposure, at the time of initial screening.

Many contacts were also confused about the way in which TB is transmitted. Over one third of contacts who did not attend their appointment did not believe TB was transmissible. Over 70% of contacts incorrectly believed that sharing utensils or other common property could transmit TB. While these beliefs could heighten their perception of risk and motivate their desire to participate in screening, fear of contagiousness may also lead to discrimination against people with TB and increase their sense of alienation from society. Simple educational interventions to explain the airborne nature of TB transmission can be incorporated into screening programs to combat this misperception.

Importantly, over half of all contacts hold the traditional Vietnamese belief that an ‘exhausted’ form of TB is non-infectious, as previously described [20]. In our focus groups some participants described this form of disease as indicating an intrinsic weakness within a family. This idea, although not biologically accurate, is understandable given the observed household clustering and known genetic risk factors for TB. These attitudes and beliefs may further exacerbate a sense of alienation among affected individuals. It is not surprising that this notion was more common among those who did not attend contact screening than among those who did attend.

Importantly, we found that 45% of Districts required contacts to travel more than 500 m from the District clinic to access external radiology facilities. This is likely to prolong each screening encounter. Similar difficulties accessing clinical services have also been shown to reduce treatment compliance in patients treated for active TB in other settings [24-26]. As over one quarter of contacts in our study lived over 10 km from the screening clinic, consideration should to be given to a more decentralised approach to screening, particularly in rural areas. Another common barrier to attendance was difficulty remembering appointment dates and times. Innovative strategies, such as scheduled mobile phone based prompts using SMS, or written follow-up reminders could provide a simple and effective solution to address this difficulty. Hence, both practical and psychosocial barriers to attendance need to be addressed by the health service, as local contact investigation strategies are developed and scaled-up in Vietnam.

Observations from this study are highly relevant to other comparable high-burden settings where contact investigation is to be implemented. National TB Programs considering implementing or scaling up contact investigation programs may benefit from conducting similar research to identify locally-relevant barriers to implementation. Surveying participants during the early stages of a newly introduced contact investigation program can provide helpful information for health managers, in order to modify screening processes, improve accessibility, refine health promotion materials and focus National TB Program staff training.

A potential limitation of this study was that contacts selected for the study had already enrolled in the main contact investigation study. For both ethical and practical reasons, it is difficult to evaluate the opinions of subjects who are unwilling to enroll in a study. While selection bias may have underestimated the prevalence of perceived barriers to accessing screening among contacts, it is likely to bias effect estimates towards the null. Nonetheless, we identified barriers using both qualitative methods (focus groups) and quantitative approaches in 12 Districts, in 6 provinces, nested within our main study. Consequently, it is likely that these represent important factors common to many potential participants in contact investigation. Hence, our results have been useful in developing resources to improve participation in the main study, and will enhance the expansion of contact investigation in Vietnam.

This study has demonstrated that contacts and patients in Vietnam commonly perceive prejudice against TB, although most participants did not feel this directly affected them. By identifying some important misunderstandings about TB transmission and pathogenesis, we have identified factors that, if addressed, may improve participation and retention in contact investigation programs.

## Conclusions

In conclusion, contact investigation is a promising intervention for improving TB case detection. It benefits both individuals and the community. By evaluating the experience of contact investigation from a range of perspectives, TB programs can better understand the way in which their policies operate in practice. These insights can improve the way in which health care is delivered in order to improve the experience of screening participants and strengthen TB control.

## Additional files

Additional file 1:
**Questionnaire for tuberculosis patients and their household contacts.**
Additional file 2: Table S1. Knowledge and attitudes among index patients.

## Abbreviations

OR: Odds ratio
TB: Tuberculosis
RCT: Randomised controlled trial
WHO: World Health Organization
