# Supplementary material for: Barriers to adherence with tuberculosis contact investigation in six provinces of Vietnam: a nested case–control study
Source: BMC Infect Dis. 2015 Feb 26;15:103. doi: 10.1186/s12879-015-0816-0 (PMC4377211; doi:10.1186/s12879-015-0816-0)
Supplement: Additional file 1: — Questionnaire for tuberculosis patients and their household contacts. [file 12879_2015_816_MOESM1_ESM.doc]

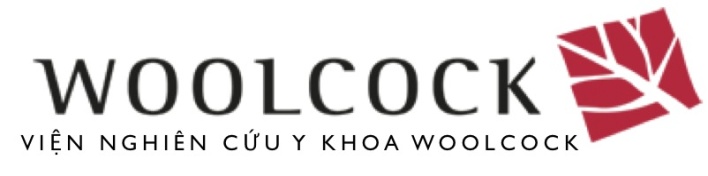


No

QUESTIONNAIRE FOR TUBERCULOSIS PATIENTS AND THEIR HOUSEHOLD CONTACTS

| Province |  |
| --- | --- |
| District |  |
| Date of interview | ............/............/2012 |
| Interviewer’s full name |  |
| Interviewee’s full name |  |
| Study number of interviewee | _ _ _ _ _ _ _ |
| If this person is a contact, are they currently a defaulter?*(A person is a defaulter if they: (a) have not attended the 6 month interview at the time of the survey, AND (b) it is more than 1 month after the date that their appointment was due).* | [ ] Yes  [ ] No |
| Did the contact agree to get involved in the survey? | a, Agree  b, Disagree  c, Can’t contact |

The study is being conducted by Woolcock Institute of Medical Research. You are invited to complete a questionnaire about knowledge, attitude and practices of TB within patients and close contacts of patients who are infected with TB. The purpose of this questionnaire is to find out your opinions about tuberculosis screening. We also want to learn about what factors might make it difficult for you to attend screening, and what can be done to improve your access to tuberculosis screening. This information will be used to help improve the way that the National Tuberculosis Program conducts screening of contacts of tuberculosis patients in the future. This will take about 10 minutes. Thank you for completing this survey!

NOTICE

1. Interviewer ask contacts sentence by sentence to avoid missing information or having wrong information
2. Selection criteria

**Index patients:**

An included index patient is a person who:

- Is enrolled in the ACT2 research (with smear positive pulmonary tuberculosis)
- Living in the same household as at least one defaulter or one non-defaulter who consents to complete a questionnaire

**Non-defaulter:**

An included non-defaulter is a person who:

- Is a household contact enrolled in the ACT2 study
- Was enrolled in the ACT2 research between March 1, 2011 and September 30, 2011
- Attends a month follow-up appointment for the research within no more than 7 months of the time of enrolment, and has an entry in the register book S2 for this follow-up appointment

**Defaulter:**

An included defaulter is a person who:

- Is a household contact enrolled in the ACT2 study
- Was enrolled in the ACT2 research between March 1, 2011 and September 30, 2011

Does not attend a month follow-up appointment for the research within no more than 7 months of the time of enrolment]

3. Sample question

| Q1 | What is the name of the disease that we are studying for this research?  *Check the one option which is most appropriate* | Tuberculosis  Malaria  Don’t know | 1  2  99 |
| --- | --- | --- | --- |

1. **BASIC INFORMATION**

| **#** | **Question** | **Answer** | **Code** |
| --- | --- | --- | --- |
| Q1.1 | What is your relationship with <name of TB patient>?  *(Check one option)* | Father/mother  Spouse  Children  Siblings  Other relative  Other (specify……………………...) | 1  2  3  4  5  88 |
| Q 1.2 | In what year were you born?  *Write YYYY, for example “1974”* |  |  |
| Q1.3 | What is the highest level of education you have finished?  *(Check one option)* | No school education  Primary school (grade 1-5)  Secondary school (Grade 6-9)  High school (Grade 10-12)  Higher education (>12)  Vocational education | 1  2  3  4  5  6 |
| Q1.4 | Currently, which of the following best describes your current main job?  *(Check one option)* | Unemployed  Student  Full-time employee (more than 7 hours per day)  Part-time employee (less than 7 hours per day)  Retiree  Freelancer (handcrafter, farmer, fish-man…)  Other (specify……………………..) | 1  2  3  4  5  6  88 |
| Q1.5 | What was the average income that you earn **each month** (including official and other sources)? | Less than 400,000 VND  From 401,000 – 520,000VND  520,000 to 5,000,000 VND  More than 5,000,000 VND  No response | 1  2  3  4  99 |
| Q1.6 | Do you or someone in your household own:  *(Check one or more options)* | a Car  a Motorbike  a Washing machine/fridge  a TV  a Radio, cassette, DVD player  a Computer | 1 or 2  1 or 2  1 or 2  1 or 2  1 or 2  1 or 2 |
| Q1.7 | How many rooms are there in the house (that you usually sleep and live in)?  (*State the number of rooms, excluding bathrooms)* | ……. |  |
| Q1.8 | Currently, do you sleep in the same room as <name of TB patient>? | Yes  No | 1  2 |

**II. KNOWLEDGE OF HOUSEHOLD CONTACT**

| **#** | **Question** | **Answer** | | **Code** | | | | | | |
| --- | --- | --- | --- | --- | --- | --- | --- | --- | --- | --- |
| ~~Q2.1~~ | I am going to read a list of possible symptoms. . For each one, please tell me if you think it is **commonly** associated with TB? For each option, say either “yes”, “no” or “I don’t know”.  *(Check one or more options)* | Cough | | Yes  1 | | No  2 | | Don’t know  99 | | |
|  |  | Fatigue | | 1 | | 2 | | 99 | | |
|  |  | Leg pain | | 1 | | 2 | | 99 | | |
|  |  | Weight loss | | 1 | | 2 | | 99 | | |
|  |  | Night sweats | | 1 | | 2 | | 99 | | |
|  |  | Increased appetite | | 1 | | 2 | | 99 | | |
|  |  | Cough up blood | | 1 | | 2 | | 99 | | |
|  |  | Chest pain | | 1 | | 2 | | 99 | | |
|  |  | Dizziness | | 1 | | 2 | | 99 | | |
|  |  | Fever | | 1 | | 2 | | 99 | | |
|  |  | Difficulty breathing | | 1 | | 2 | | 99 | | |
| Q 2.2 | **Which of the following activities causes a person with TB to spread the infection to others? For each option, state “Yes”, “No”, or “Don’t know”**  *(Check one or more options)* | | | **Response** | | | | | | |
|  |  |  |  | **Yes** | | | **No** | | | **Don’t know** |
|  | When patient sneezes | | | 1 | | | 2 | | | 99 |
|  | When patient talks | | | 1 | | | 2 | | | 99 |
|  | When eating and drinking using the same utensils (such as bowl, chopschecks, glass) as the person with TB | | | 1 | | | 2 | | | 99 |
|  | When sleeping in the same room as the person with TB | | | 1 | | | 2 | | | 99 |
|  | When using same belongings (towels, clothes, napkins) | | | 1 | | | 2 | | | 99 |
|  | When hugging or kissing the person with TB | | | 1 | | | 2 | | | 99 |
|  | When using same toilet | | | 1 | | | 2 | | | 99 |
|  | When having sexual intercourse with the person with TB | | | 1 | | | 2 | | | 99 |
| Q 2.3 | **Now I am going to read some statements about natural history of TB. Some of them are true and some are not true. For each option, say if it is “true”, “false” or you “Don’t know”.**  *(Check one or more options)* | | | | | | | | | |
|  | **Statement** | | **Response** | | | | | | | |
|  |  |  | **True** | | **False** | | | | **Don’t know** | |
|  | TB is caused by an infection | | 1 | | 2 | | | | 99 | |
|  | TB is caused by living in an unhygienic environment | | 1 | | 2 | | | | 99 | |
|  | Tuberculosis is acquired by being inherited from your parents | | 1 | | 2 | | | | 99 | |
|  | Some people have exhausted TB which cannot be spread from person to person | | 1 | | 2 | | | | 99 | |
|  | Tobacco and bamboo bong smoking increases susceptibility to TB | | 1 | | 2 | | | | 99 | |
|  | Children are at a greater risk of developing TB than adults | | 1 | | 2 | | | | 99 | |
|  | People with HIV/AIDS have a higher risk of developing TB than a person without HIV | | 1 | | 2 | | | | 99 | |
|  | Pregnant women are at a greater risk of developing TB than people who are not pregnant | | 1 | | 2 | | | | 99 | |
|  | People with weakened immune system due to chronic disease such as diabetes are at a higher risk of developing TB | | 1 | | 2 | | | | 99 | |
|  | People with poor nutrition have a higher risk of developing TB | | 1 | | 2 | | | | 99 | |
|  | TB can be completely cured if a patient takes treatment. | | 1 | | 2 | | | | 99 | |
|  | Traditional medicine can be used to cure tuberculosis | | 1 | | 2 | | | | 99 | |
|  | Western medicine can be used to cure tuberculosis | | 1 | | 2 | | | | 99 | |
|  | TB can severely affect your health | | 1 | | 2 | | | | 99 | |

III ATTITUDE OF HOUSEHOLD CONTACT TOWARD SCREENING PROGRAMME

| **#** | **Question** | **Answer** | **Code** | |
| --- | --- | --- | --- | --- |
| Q 3.1 | Do you think that there is discrimination against people with TB?  *(Check one option)* | Yes  No  Don’t know | 1  2  88 | |
| Q 3.2 | How would you rate your risk of developing TB compared to a member of the general population?  *(Check one option)* | Much lower risk  Lower risk  Similar risk  Higher risk  Much higher risk | 1  2  3  4  5 | |
| Q 3.3 | For defaulters only:  **State whether you agree or disagree with each of the following statements?**  *(Check one or more options)* | **I did not attend follow-up because:**  The distance to travel from my home to preventative district health center is too far | **Agree**  1 | **Disagree**  2 |
|  |  | I worried about the harmful effect of an Xray | 1 | 2 |
|  |  | I wanted to be examined in a private health clinic instead | 1 | 2 |
|  |  | I am worried about discrimination from other people toward myself and my family | 1 | 2 |
|  |  | The patient in my household recovered, so it is unnecessary to follow up the screening anymore | 1 | 2 |
|  |  | I didn’t remember the appointment date | 1 | 2 |
|  |  | The initial contact investigation was negative, therefore no reason to attend follow up | 1 | 2 |
|  |  | It is time-consuming. It was difficult to get time off work or study | 1 | 2 |
|  |  | I was pregnant | 1 | 2 |
|  |  |  |  |  |
| Q 3.4 | I am going to read a statement, and I would like you to choose one option that reflects your opinion.  “Overall, the TB screening program is beneficial for me and my family?”  *(Check one option)* | Strongly disagree  Disagree  Neither agree nor disagree  Agree  Strongly agree | 1  2  3  4  5 | |

**IV. PRACTICE OF HOUSEHOLD CONTACTS IN SCREENING PROGRAMME**

| **#** | **Question** | **Answer** | **Code** |
| --- | --- | --- | --- |

**V. ACCESSIBILITY OF HOUSEHOLD CONTACTS IN SCREENING PROGRAMME**

| **#** | **Question** | **Answer** | **Code** |
| --- | --- | --- | --- |
| Q 5.1 | How did the health care worker inform you about the 6 month appointment? | Telephone  Visit to my house  Sent a letter to my house  They did not contact me to remind me  Other (specify……………………….) | 1  2  3  4  88 |
| Q 5.2 | How far do you live from the District clinic?  (*Check the one answer that is most suitable)* | Less than 2 km from the Clinic  Between 2 km and 9km from the Clinic  From 10 km to 15 km  16km or above | 1  2  3  4 |
| Q 5.3 | How would you usually get to the district health clinic?  (*Check the one answer that is most suitable)* | Walking  Bicycle  Motorbike  Bus  Other (specify……………………….) | 1  2  3  4  88 |
| Q 5.4 | How long did you have to wait at the District Clinic before you have completed screening?  (*Check the one answer that is most suitable)* | Less than 1 hour  From 1 hour to 2 hours  Above 2 hours  Don’t remember | 1  2  3  4 |
| Q 5.5 | Have any of the following people talked to you about your risk of getting TB in the last 6 months?  *(Check one or more options)* | Village health worker  Communal health staff  District health staff  Other health care staff  Patient with tuberculosis  Mass organization (women’s, youth’s union) | 1 or 2  1 or 2  1 or 2  1 or 2  1 or 2  1 or 2 |
