# Supplementary material for: Barriers to adherence with tuberculosis contact investigation in six provinces of Vietnam: a nested case–control study
Source: BMC Infect Dis. 2015 Feb 26;15:103. doi: 10.1186/s12879-015-0816-0 (PMC4377211; doi:10.1186/s12879-015-0816-0)
Supplement: Additional file 2: Table S1. — Knowledge and attitudes among index patients. [file 12879_2015_816_MOESM2_ESM.docx]

**Additional file 2: Table S1: Knowledge and attitudes among index patients**

|  | **Index patients of**  **cases** | | **Index patients of controls** | | **Odds of contact**  **non-attendance** | |
| --- | --- | --- | --- | --- | --- | --- |
| **Characteristic** | **n** | (% / IQR) | **n** | (% / IQR) | **OR** | **95% CI** |
| **Total** | **62** |  | **108** |  |  |  |
| **Attitudes towards TB** |  |  |  |  |  |  |
| Perceive discrimination against TB from outside the family**¶** | 31 | (50%) | 48 | (47%) | 1.15 | (0.61 -2.15) |
| Believe that own risk of TB as being higher than the general population | 51 | (80%) | 87 | (80%) | 1.0 | (0.47 -2.16) |
| Believe TB screening is beneficial for their family | 60 | (94%) | 100 | (93%) | 1.2 | (0.35 -4.16) |
| **Knowledge and attitudes about TB** |  |  |  |  |  |  |
| TB can be transmitted by |  |  |  |  |  |  |
| Sneezing | 23 | (37%) | 43 | (40%) | 0.91 | (0.48 -1.74) |
| Talking | 47 | (76%) | 91 | (84%) | 0.59 | (0.27 -1.29) |
| Sharing utensils | 50 | (81%) | 92 | (85%) | 0.73 | (0.32 -1.67) |
| Sleeping in the same bedroom | 46 | (74%) | 85 | (79%) | 0.79 | (0.38 -1.64) |
| Sharing towels, clothes or napkins | 36 | (58%) | 75 | (69%) | 0.62 | (0.32 -1.18) |
| When hugging or kissing | 51 | (82%) | 90 | (83%) | 0.94 | (0.41 -2.14) |
| Sharing the same toilet | 19 | (31%) | 34 | (31%) | 0.99 | (0.5 -1.95) |
| When having sexual intercourse | 9 | (15%) | 12 | (11%) | 1.34 | (0.53 -3.4) |
| TB is caused by: |  |  |  |  |  |  |
| An infectious organism | 34 | (55%) | 83 | (77%) | 0.37 | (0.19 -0.72) |
| Living in an unhygienic environment | 52 | (84%) | 91 | (84%) | 0.98 | (0.42 -2.3) |
| Inheriting the disease from your parents | 18 | (29%) | 23 | (21%) | 1.58 | (0.77 -3.25) |
| A form of ‘exhausted TB’ which is not transmissible | 33 | (53%) | 52 | (48%) | 1.25 | (0.67 -2.34) |
| The following people have a higher risk of developing TB |  |  |  |  |  |  |
| Tobacco and bamboo bong smokers | 61 | (98%) | 102 | (94%) | 3.62 | (0.43 -30.82) |
| Children | 52 | (84%) | 84 | (78%) | 1.5 | (0.67 -3.4) |
| Pregnant women | 53 | (85%) | 68 | (64%) | **3.43** | **(1.53 -7.7)** |
| People with weakened immune systems such as diabetes | 59 | (95%) | 87 | (81%) | **4.8** | **(1.37 -16.83)** |
| People with poor nutrition | 59 | (95%) | 101 | (94%) | 1.38 | (0.34 -5.53) |
| Treatment and cure of TB: |  |  |  |  |  |  |
| TB can be completely cured if a person takes treatment | 61 | (98%) | 107 | (100%) | na |  |
| Traditional medicine can cure TB* | 7 | (11%) | 7 | (7%) | 1.8 | (0.6 -5.4) |
| Western medicine can cure TB | 61 | (98%) | 105 | (97%) | 1.76 | (0.18 -17.29) |
| TB can severely affect your health | 52 | (84%) | 102 | (94%) | 0.31 | (0.11 -0.9) |

OR - Unadjusted odds ratio. **^¶^**102 control responses given. *103 control responses

given.
